# Supplementary material for: Relationships Between Misinformation Variables and Nutritional Health Strategies: A Scoping Review
Source: Int J Environ Res Public Health. 2025 Jun 3;22(6):891. doi: 10.3390/ijerph22060891 (PMC12192748; doi:10.3390/ijerph22060891)
Supplement: Supplementary file 1 [file ijerph-22-00891-s001.zip › ijerph-3538534-supplementary.pdf]

Title, Date, Country/ Region	**Author**	**Study aim**	**Study design**	**Population**	**Sample size**	**Intervention**	**Source of potential misinformation**	**Key Findings**	**Limitations/Gap in the Research**
Nutripedia: The Fight against the Fake News in Nutrition during Pregnancy and Early Life, 2021, Italy (Online)	Verduci E, Vizzuso S, Frassinetti A, Mariotti L, Del Torto A, Fiore G, Marconi A, Zuccotti GV.	Introduced novel tools of e-health communicat ion with the scope of counteractin g, nutritional fake news concerning pregnancy	Interventional study, using e-health communicatio n tools (both qual & quant)	Parents	Website engagement: 220,000 total views<br>Social Media Reach: 9 million<br>Chatbot: 14,698 downloads	Promoted new e-health communication tools i.e., Nutripedia website and Chatbot app via nongovernment al organization outreach, digital media sources and scientific society conferences. Delivered general population advice as well as individualized information and intervention. Information focused on nutritional knowledge (preconception, pregnancy, children to 3 years).	Fake news concerning pregnancy and first 1000 days of life. Lack of support for new parents and gaps in nutritional knowledge could leave them susceptible to inaccurate online information. Parents' nutritional knowledge can also be affected by online tools, social environment, peers' advice, medical assistance, personal convictions.	With an abundance of information and resources being available to parents, it made it harder for them to mobilize their knowledge. This lead to difficulties for parents in identifying false information.	Future research could investigate the effectiveness of this type of intervention while focusing on different indicators such as: changes in parental attitudes and behavioural intentions, or usability and reliability of communication tools by paediatricians
Nutrition labels' strengths & weaknesse s and strategies for improving their use in Iran: A qualitative	Seyedhamze h S, Nedjat S, Shakibazade h E, Doustmoha mmadian A, Hosseini H, Dorosty Motlagh A.	Explained the strengths and weaknesses of the Traffic Light Label, and Nutrition Fact Label and the strategies for improving	Qualitative study - Focus Group Discussions	Mothers, food quality control expert (FQC), nutritionist s, and food industry experts	Mothers, n = 63<br>Children, n = 63	Traffic light label (TLL) and nutrition facts label (NFL)	Mothers: Lack of trust in information provided by the manufacturers, incompatibility with the culture Otherwise, labels misleading the consumer,	The main challenge with misinformati on when it comes to the implementat ion of the labels is that the consumers	Limitations of the study includes conflicts of interest from the food quality control experts and not enough policymakers being interviewed in the research

study, 2020, Iran		their use in Iran					discrepancies in colouring reported by different laboratories, different approaches adopted by regulatory experts, ambiguous, lack of supervision and consistency.	are not well aquainted with these labels, which leads to them being misinformed. Another important weakness highlighted by the consumers is ambiguous/high amount of information provided which can make it difficult to understand the main message behind these labels.	
TikTok as an Educational Tool for Kidney Stone Prevention, 2023, USA	Salka, B; Aljamal, M; Almsaddi, F; Kaakarli, H; Nesi, L; Lim, K	Evaluated the reach and quality of kidney stone prevention information on TikTok.	Cross-sectional analysis	TikTok users	Videos, English, related to hashtag/topic, with >1000 views, 87 videos, 8.75million views	TikTok campaign #kidneystoneprevention	The majority of the TikTok videos, which did not meet American Urological Association recommendations for diet therapies in stone prevention.	The majority of videos on TikTok failed to match recommendations regarding diet therapies for kidney stone prevention, as shown by a low DISCERN score. The authors recommend	The study was not always able to identify when the video was coming from a physician or a non-physician; in cases where the information could not be obtained, the user was classed as non-physician.The study did not analyse how viewers interpret or act upon the

								ed social media engagement by urologists and health organizations to improve education and decrease misinformation as the average DISCERN score for physicians was much higher than that of non-physicians.	information in these videos, which may be an interesting aspect to analyse.
Transferability of the Mediterranean diet to non-Mediterranean countries. What is and what is not the Mediterranean diet, 2017, USA	Martínez-González M.Á., Hershey M.S., Zazpe I., Trichopoulou A.	Presented strategies necessary for promoting MedDiet to non Med context i.e., Americans	Cumulative meta-analysis	Those at risk for cardiovascular disease	27 articles	MedDiet	Popular definitions of MedDiet were not in line with traditional diet, leding to myths/misconceptions and promotion of foods that are not in line with MedDiet benefits.	In order to effectively transfer the Mediterranean diet to non-Mediterranean countries the misinformation regarding the diet needs to be addressed by effecteviely defining what is and what isn't included in the diet. More efforts should also be made to	More research on the transferability and effectiveness of the MedDiet to the US and the benefits in non-Mediterranean population is needed. The authors suggested a randomized intervention study to obtain this evidence.

								adapt the diet to the local cultures.	
Translating Evidence-Based Program Recommendations into Action: The Design, Testing, and Scaling Up of the Behaviour Change Strategy EsIAN in Mexico, 2019, Mexico	Bonvecchio Arenas A, González W, Théodore FL, Lozada-Tequeanes AL, Garcia-Guerra A, Alvarado R, Fernández-Gaxiola AC, Rawlinson CJ, de la Vega AV, Neufeld LM.	Described the process and evi-dence-based ap-proach used to design and rollout the EsIAN at scale. Focused on the behavior change commu-nication compo-nent	Mixed methods	Mothers/caregivers of chilgren aged < 5 years, health care providers, experts	Up to n=1387 depending on the phase of the study	Integrated strategy for attention to nutrition, specifically the implementation of the behavior change communication (BCC) strategy component	Mothers: misconceptions during pregnancy and breastfeeding e.g., 'they should eat for two during pregnancy'. Cultural beliefs e.g., early introduction of foods and liquids could stem from mothers, mothers-in-law, fathers, grandparents. Additionally, primary health care providers also sometimes lacked the knowledge and skills concerning nutrition advice during pregnancy and lactation. Recommendations did not always align	The EsIAN strategy alone was insufficient to address the double burden of malnutrition and misinformation and multisectional engagements are needed for significant impact. The study addressed that misinformation is a real issue and a real burden and a good first step in addressing these issues is through nutritional campaigns.	The lack of comparison group in the feasibility study could yield a less robust evaluation. Time restrictions on the implementation. Effects of the implementation could've been explored further by using more indicators in the study.

							with global and national recommendations for mothers/caregivers.		
Twelve-Month Efficacy of an Obesity Prevention Program Targeting Hispanic Families With Preschoolers From Low-Income Backgrounds, 2021, USA	Hughes, SO; Power, TG; Beck, AD; Betz, D; Goodell, LS; Hopwood, V; Jaramillo, JA; Lanigan, J; Martinez, AD; Micheli, N; Guerrero, YO; Overath, I; Parker, L; Ramos, G; Thompson, YP; Papaioannou, MA; Johnson, SL	Assessed effects of an obesity prevention program which promoted eating self-regulation and healthy preferences	Randomized control trial	Hispanic preschool children (parents + families with low incomes)	Families recruited from *Head Start across 2 sites, 255 families randomized (prevention n = 136; control n=119)*	SEEDS (Strategies for Effective Eating Development) obesity prevention programme. Curriculum aiming to change feeding knowledge/practices/styles (parent); BMI percentile, eating self-regulation, trying new foods, and fruit/vegetable consumption (child)	Mothers were sometimes a source of feeding misconceptions.	The prevention program showed changes in parental feeding behaviours, increased knowledge of best feeding practices, reduced feeding misconceptions and less uninvolved feeding styles. As a result, researchers believed that changes observed in child weight status are potentially a result of changes in maternal feeding practices	Measures and indicators that are more sensitive to children from different cultures and socioeconomic statuses need to be developed in order for more accurate research and results in these contexts. Further research could also include better measurements of child eating behaviours and the effects of implementation on such.

								and knowledge.	
"Babies know the facts about folic": A behavioural change campaign utilising digital and social media, 2016, Ireland (Hybrid)	Flaherty et al.	Assessed if the "Babies Know the Facts about Folic" campaign changed women's knowledge, attitudes and behavior towards folic acid supplements	Online survey was conducted pre- and post-campaign. Home face-to-face interviews were conducted three months after the campaign.	Women of a childbearing age who are sexually active and could become pregnant	online survey: n = 656/738 interviews: n = 424	"Safefood" launched a social and digital media campaign in 2015	Misconceptions of women concerning the supplementation of folic acid prior and during pregnancy that could lead to low consumption of the supplement despite benefits.	The campaign was successful in changing individual's knowledge, attitudes and behaviour in relation to folic acid supplements thus highlighting a potential decrease in misinformation. Study authors concluded that results highlight the effectiveness of social media and digital media strategies for changes in health behaviour.	The study is relatively short-termed, therefore the long term effects of the implementation are unknown. There is also a lack of control group to assess the true effectiveness of the implementation.

Human-centred designed communication tools for obesity prevention in early life, 2023, USA (Indianapolis)	Cheng et al.	To develop tools to ease the provider-parent communication about obesity prevention in a paediatric setting	Co-design workshops with parents and paediatricians	Parents and caregivers of children aged 0-24 months and paediatricians	13 parents (11 mothers, 2 fathers<br>13 paediatricians	Activities based on a human-centered design, involving parents and pediatricians. Activity 1: discussed communication barriers/facilitators on child obesity prevention. Activity 2: created visual aids to facilitate the parent-provider communication.	There were knowledge barriers and misconceptions relating to infant feeding. In a needs assessment of parents of children aged 0-24 months only a few parents viewed obesity in early life as a health issue.	Two tools informed by stakeholders were created to tackle common misconceptions about health behaviours in children ages 0 to 24 months. The purpose of the tools was to provide easy guidelines about healthy-weight promoting behaviours . Guidelines addressing common misconceptions regarding feeding, sleep, and exercise were included in these tools.	Small sample size, low participation from minoritized communities, low participation from low income households. Not all parents completed activities. Stress related confounders such as COVID were not assessed. Feasibility of integrating the tool was not assessed.

Perspectives on healthy eating practices and acceptance of WIC-approved foods among parents of young children enrolled in WIC, 2023, USA	Hammad and Kay	Analyzed what parents consider as healthy food habits, acceptance of certain food categories and the acknowledgement towards digital tools to improve diet quality	Qualitative interviews	Parents or caregivers of children aged 0-2 years who received benefits from the "Special Supplement Nutrition Program for Women, Infants, and Children", had a cell phone and spoke English	n = 13	The "Special Supplement Nutrition Program for Women, Infants, and Children" aimed to improve nutritional intake of low-income mothers and their 0-5 years old children. Food packages containing vouchers for nutrient rich foods were distributed to the intervention's beneficiaries. However, the program has lately faced a decrease in participation, retention, and the redemption of food packages.	Mothers had different definitions of healthy versus unhealthy eating, possibly due to the variety of sources consulted to find nutrition information e.g., social media, Google, YouTube, nutritionist from the intervention, friends, family, partners, cooking shows, research, own common sense.	The study highlighted the importance of adequate communication with target community before the implementation of an intervention to ensure relevance of the intervention. The study concluded that based on the findings, barriers could be reduced by tailoring educational material to these topics, raising awareness about these topics and making structural changes.	Small sample size of 13 offered less generalizable results. Most participants came from urban settings; immigrant groups were not represented.

Busting the Baby Teeth Myth and Increasing Children's Consumption of Tap Water: Building Public Will for Children's Oral Health in Colorado, 2017, USA	Hornsby et al.	Evaluated if a communication campaign could change behavior to limit children's fruit juice consumption and increase tap water consumption to improve oral health.	Pre- and post-campaign surveys applying quantitative methods	Low-income families living in Colorado who have a child between 6 months and 6 years of age	n = 603/600	"Cavities Get Around" is a statewide communication campaign including television and radio advertisements, social media, health promoters, educational programs, text messaging and community partnerships to increase children's consumption of tap water and decrease consumption of fruit juice and other sugary drinks.	Fruit juice was commonly regarded as healthy, although it contains a lot of sugar and therefore increases the risk for tooth decay in infants and children. Moreover, many parents underestimated the importance of baby teeth, regarding them as less important than adult teeth. Many parents did not know that cavities could spread from baby to adult teeth.	Reported improved perception of the importance of children drinking tap water, increase in children's consumption of tap water, decrease in the perception of fruit juice consumption as healthy for children, and reported decrease in children's consumption of fruit juice.	Although the random sampling methods were similar for both surveys, the respondents were different making it impossible to know if attitudes and behaviours change in specific families or in individuals. Responses were self-reported and unverified, impact of societal trends were also unaccounted for.
Effectiveness of health promotion regarding diet and physical activity among Nepalese mothers and their young children: The Heart-health Associated Research,	Oli et al.	Assessed the effectiveness of a health promotion intervention on mothers' knowledge, attitude and practice and their children's behavior regarding diet and physical activity.	Baseline and follow-up survey (quantitative methods)	Mothers of children aged 1-9 living in one of the two neighbouring villages Duwakot (intervention area) or Jhaukhel (control area)	n = 323 mothers completed round 1 of the intervention n = 105 mothers completed round 2 of the intervention	The "Heart-health Associated Research, Dissemination and Intervention in the Community", (HARDIC): a community-based health education program designed to improve diet and physical activity as part	Widespread misconceptions by mothers who did not always understand the composition of healthy food. Additionally, the mothers believed to have only little control over their children's eating habits.	KAP (knowledge-attitude-practice) scores in the intervention village increased significantly from baseline to follow-up. DiD(differences-in-differences) analysis showed	Trial was limited to only two rounds of intervention, follow-up study was conducted 3 months after the intervention and therefore was only able to demonstrate short-term impact. Possibility of bias in the interpretation of the results as

Dissemination, and Intervention in the Community, 2019, Nepal (HARDIC) trial						of cardiovascular health promotion. 47 peer mothers were trained to conduct five education classes for around 10 fellow mothers each.		significant improvement in children's behaviour regarding diet and physical activity, however median score for children's behaviour was the same in the intervention at baseline and follow-up	children's behaviours were assessed by their mother's perception and not directly by them. Potential of other confounding biases which may affect the estimated impact of the program
Effectiveness of nutrition education in improving fruit and vegetable consumption among selected college students in urban Puducherry, South India. A pre-post intervention study, 2020, India (Puducherry)	Patel N., Lakshminarayanan S., Olickal J.J.	Evaluated the effectiveness of nutrition education in improving the daily intake of fruit and vegetable servings, and stage of behaviour change among college students	Randomized control trial	Urban college students	n = 150	Intervention Group => 30 minutes of nutrition education programme Control Group => pamphlets regarding healthy dietary intake	Initially, less knowledge regarding portion sizes, average daily servings of fruit & vegetable intake and stage of behaviour change among college students	The strategies used for intervention significantly improved the correct knowledge regarding the number of servings of fruits and vegetables consumed per day. The knowledge regarding the portion size also increased significantly in the intervention group. Study	The study period was limited to four weeks, the participants could not be followed up for long term sustainable behaviour change. There may have been social desirability bias which could have led to over reporting of fruit and vegetable consumption

								concluded that nutrition education is an effective tool for promoting healthy eating habits.	
Gluten-free diet on video platforms: Retrospective infodemiology study, 2024, China	Ye, C; Fang, YH; Lian, YY; He, YN	Examined the trends, content, and quality of information on two social media platforms	Mixed Methods: Mann-Kendall tests, DISCERN, HONcode	Videos using #GFD on TikTok and BiliBili	TikTok, n = 49 BiliBili, n = 86	Gluten-Free diet videos	Quality of health information videos on Chinese video platforms were poor. The majority of them were not rigorous enough, containing misleading messaging that could promote e.g., unproven treatments with no discussion of possible side effects.	There is an increase in interest regarding gluten-free diet videos in China however the poor quality of these videos promotes misinformation and disinformation. The authors suggested that policy development of video platforms and education for health professionals on making videos are necessary to avoid these issues.	There were some limitations in assessing the quality of videos and their content. Number of engagements were analysed but there was no analysis as to whether these were positive or negative engagements.

Influence of Teachers' Personal Health Behaviours on Operationalizing Obesity Prevention Policy in Head Start Preschools: A Project of the Children's Healthy Living Program (CHL), 2016, Hawaii	Esquivel M.K., Nigg C.R., Fialkowski M.K., Braun K.L., Li F., Novotny R.	Quantified the Head Start (HS) teacher mediating and moderating influence on the effect of a wellness policy intervention.	Intervention trial within a larger randomized community trial	Teachers from 23 Head Start (HS) classrooms	n=46	Seven-month multi-component intervention with policy changes to food served and service style, initiatives for employee wellness, classroom activities for preschoolers promoting physical activity (PA) and healthy eating, and training and technical assistance.	Knowledge, beliefs, priorities, and misconceptions around child nutrition among teachers could influence child nutrition and obesity.	Study findings suggested positive effects of a policy intervention on the HeadStart campaign. Also demonstrated the impact of teacher's personal health behaviours on child nutrition and the success of a classroom based intervention.	Reliance on single assessments of classroom observations and on self-reported changes in behaviours is limiting. The authors recommended to repeat an alternative assessment in future investigations. Sample was limited to one geographic region of Hawaii and may not be generalizable.

Table S1: Complete Characteristics of the Included Studies.
